# Oral Medicine in Latin America and the Caribbean: A comprehensive survey of recognition, training, and practice

**DOI:** 10.4317/medoral.26944

**Published:** 2025-04-06

**Authors:** Cristina Saldivia-Siracusa, Leonor Victoria González-Pérez, César Rivera, Daniela Porras Guevara, Daymar Aviles, Eduardo David Piemonte, Efrain Cima Garcia, Elisa Contreras-Vidaurre, Gabriela Anaya-Saavedra, Gennisa Gutierrez Pérez, Gloria J Álvarez Gómez, Itza Rios, Leira Patricia Solís Espinal, Nathalie Amaya Londoño, Roberto Anaximandro Garcia Rejas, Rodolfo Epifanio, Sergio Castro Mora, Susana Vázquez Celhay, Marcio Ajudarte Lopes, Alan Roger dos Santos-Silva

**Affiliations:** 1 Oral Diagnosis Department, Semiology and Oral Pathology Areas, Piracicaba Dental School, University of Campinas (UNICAMP), Piracicaba, Sao Paulo, Brazil; 2 Laboratory of Immunodetection and Bioanalysis. Investigation Group POPCAD, Faculty of Dentistry, University of Antioquia, Medellín, Colombia; 3 Department of Stomatology, University of Talca (UTALCA), Talca, Maule Region, Chile; 4 School of Dentistry, Santa Maria University (USM), Caracas, Venezuela; 5 Dentistry Research Institute, Central University of Venezuela (UCV), Caracas, Venezuela; 6 Oral Medicine Department, Faculty of Dentistry, National University of Córdoba (UNC), Córdoba, Argentina; 7 Corozal Community Hospital, Corozal, Belize; 8 Oral Medicine Department, School of Dentistry, Francisco Marroquín University, Guatemala City, Guatemala; 9 Oral Pathology and Medicine Postgraduate Program, Metropolitan Autonomous University, Mexico City, Mexico; 10 The Dental Centre, St Clair, Trinidad and Tobago; 11 Faculty of Dentistry, University of Antioquia, Hospital San Vicente Fundación, Oral and Maxillofacial Surgery Service, Medellín, Colombia; 12 Health Ministry, University of Panama, Panama City, Panama; 13 School of Dentistry, Santo Domingo Autonomous University, Santo Domingo, Dominican Republic; 14 Oral Pathology Department, Faculty of Dentistry, University of Buenos Aires, Argentina; 15 School of Dentistry, del Valle University, Bolivia; 16 Stomatology Department, School of Dentistry, Panamá University, Panama City, Panama; 17 School of Dentistry, Costa Rica University, San Jose, Costa Rica; 18 School of Dentistry, University of the Republic, Montevideo, Uruguay

## Abstract

**Background:**

This study aimed to investigate the scope of training and practice in Oral Medicine in Latin American and Caribbean countries. It explored legal, professional, and academic scope of regional OM practice, as well as current challenges perceived by experts in the field.

**Material and Methods:**

We employed an observational, cross-sectional approach, utilizing a self-administered questionnaire delivered through the REDCap web platform.

**Results:**

Oral Medicine is officially recognized as a dental specialty in 66.7% of Latin American and Caribbean countries, and 66.7% countries recognize it as a standalone field, separate from Oral Pathology. Additionally, 23.8% of the surveyed countries have national postgraduate Oral Medicine programs. Nearly half (47.6%) of the countries lack specific regulations, and there is significant variation in understanding clinical competencies. Private practice emerged as the dominant field of work for Oral Medicine practitioners. Notably, 90.5% of respondents identified the lack of recognition by multidisciplinary teams as a significant barrier to the practice.

**Conclusions:**

This study provides information on the current landscape of Oral Medicine practice in Latin American and Caribbean countries. It highlights disparities in recognition, regulation, and performance of the specialty. These findings call for international initiatives aimed at enhancing training pathways, scope of practice and the impact of Oral Medicine in the region.

** Key words:**Oral medicine, stomatology, Latin America, Latin America and the Caribbean, specialty boards, professional practice, training programs.

## Introduction

Oral Medicine (OM), also known as Stomatology in Latin America and specific Southern European regions (1), focuses on the diagnosis and non-surgical management of patients afflicted by conditions affecting the oral and maxillofacial region (2). Moreover, it plays an essential role in delivering comprehensive oral healthcare to patients with systemic diseases (3,4), providing a crucial interphase between dentistry and medicine with growing demand due to ongoing increase in aging populations, the use of innovative treatments with oral repercussions, and the shift toward nonsurgical, health-promoting dental practices (5,6).

OM has steadily developed as a recognized area of practice within science and clinical care (1,2,7). Yet, the worldwide need for OM care is well documented (1,8,9). Despite its paramount significance, there is a noTable lack of global standardization, recognition, and training in this domain (7,10), resulting in far-reaching implications for the healthcare system, patient care, and professional development (11,12).

Latin America and the Caribbean (LAC) represent a vast region with diverse geographical, cultural, governmental, and social qualities (13). As of today, there is few studies contemplating LAC when assessing the status of OM scope, training, and practice (2,14). To enhance further growth of the OM specialty, the need to delineate optimal global and regional evidence about this matter is recognized (4), especially considering understudied regions.

To deepen our understanding, a questionnaire was used to obtain data about the LAC landscape of OM, exploring official recognition and practice regulations, existence of OM associations, definition of OM competencies, policies for quality assurance and recertification, current work fields, professional performance, and remuneration. Also, we assessed the availability of training programs, funding resources for OM training, and challenges perceived by experts in the field regarding OM practice. By providing a comprehensive portrayal of the current profile of OM practitioners across LAC, this study aims to report useful information and offer insights for future global decisions regarding educational and professional advancement within the field of OM.

## Material and Methods

- Study design

This was a cross-sectional, observational study based on the application of a survey, conducted in adherence to the Declaration of Helsinki. The study protocol was approved by the Research Ethics Committee of Piracicaba Dental School, University of Campinas (FOP-UNICAMP) (approval number: 55115521.3.0000.5418). For this study, all respondents provided written informed consent.

- Study intervention

The instrument utilized for quantitative data collection in this study was a self-administered online questionnaire, developed and managed using the REDCap electronic data capture tool hosted at FOP-UNICAMP (Version 13.8.1, Vanderbilt University, Nashville, Tennessee, USA) (15,16). REDCAP is a secure, web-based software platform useful for conducting survey-based research investigations. The survey was made available in English and Spanish in efforts to standardize this instrument and to encompass broad participant engagement. Given the academic background of potential participants, we expected that these languages would cover a wide enough audience. A forward translation method was performed by two independent bilingual researchers. A third naïve translator, unrelated to the research, produced a third translation. Discrepancies between the three translators were discussed and resolved by consensus. To assess validity, interpretation and practicability, the survey was pilot tested internally within members of this multinational team and volunteer senior OM and oral pathology (OP) practitioners of the authors’ institutions (17). Nevertheless, investigators were available to potentially adapt the questionnaire for the actual responders, if needed. The survey link was distributed to the targeted participants via email between February 22 to October 10, 2022. To enhance participant recruitment, two subsequent reminder emails were sent. The questionnaire, available at https://redcap.link/yken54n1, encompassed 31 questions categorized into four distinct domains: (1) Participant demographic information (8 questions); (2) Specialty characterization (13 questions); (3) Academic training (6 questions); and (4) Professional practice (4 questions).

- Eligibility criteria

A LAC panel was stablished (according to Pan American Health Organization WHO Countries and Centers, https://www.paho.org/en/countries-and-centers), comprising experts in the field of OM (Supplement 1). A total of 33 nations were assessed for recruitment of at least one specialist per country.

The following criteria were used to define an OM expert: dentists prepared with OM-focused postgraduate training (including residencies, specialties, diplomas, master’s degrees, and/or doctorates), and, in countries where participants with these requirements could not be found, broader consideration was extended to encompass professionals specialized in related domains, such as OP and oral and maxillofacial surgery, among others. OM experts were contacted via e-mail after consulting national/international OM organizations and senior and mid-career OM practitioners in regions where such organizations were absent. Exclusion criteria fit for those participants with previous but not current professional performance in LAC. Incomplete questionnaires were disregarded from further analysis. A non-probabilistic purposive sample was used.

- Data analysis

Two researchers (CSS and LVGP) conducted data organization and analysis. Disagreements were resolved through direct communication with the survey respondents. In instances where participants could not distinguish between OM and OP, we accepted provided responses that encompassed both fields, and those responses were subjected to comprehensive analysis. Obtained data was extracted and tabulated using Microsoft Excel® application software version 2110 (Microsoft Office LTSC Professional Plus 2021 for Windows, Microsoft Corp., Washington, USA). A descriptive and quantitative analysis was performed to report categorical and continuous variables, using mean, median, range, and frequency percentage values. All analyses were performed using SPSS version 25 (SPSS Inc., Chicago, USA).

## Results

**Figure 1:**
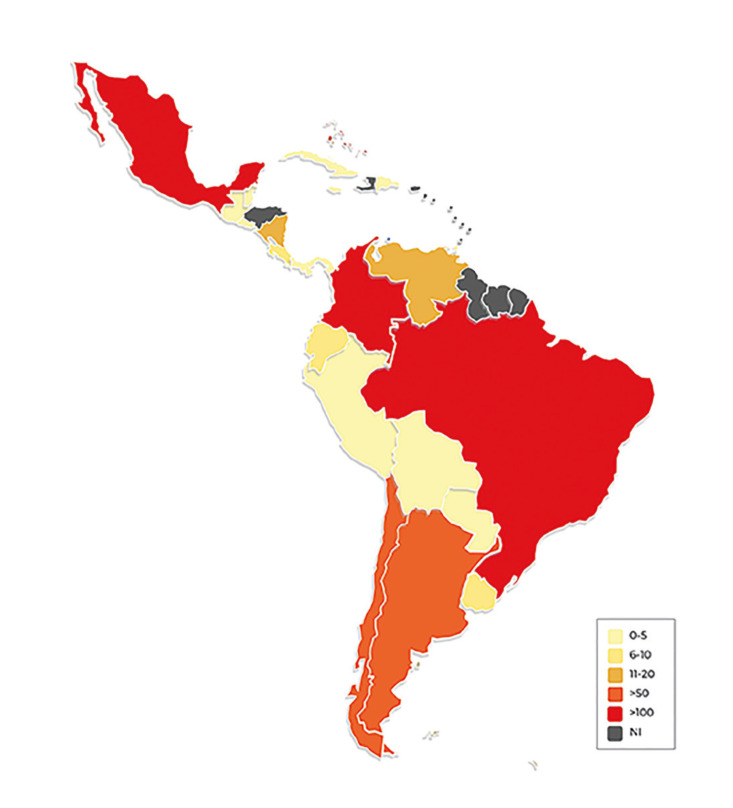
Heatmap showing distribution and number of Oral Medicine practitioners by participant country.

Within the study’s timeframe (February 22 to October 10, 2022), we contacted a total of 33 potential participants and received a total of 24 responses. Three of those were incomplete and therefore excluded from the results. Twenty-one participants fully completed the survey, each one from a different LAC country (Fig. 1). A comprehensive list of the participants and detailed listing of the LAC countries that did not partake in the survey can be found in Supplement 2.

Demographic aspects

Table 1 summarizes the demographic and academic characteristics of the 21 study participants. Gender distribution was balanced, with 10 (47.6%) females and 11 (52.4%) males. The participants’ ages ranged from 32 to 76 years, with a mean age of 46.9 years. The majority (90.5%) are employed in their home countries, while a minority (9.5%) work in other countries.

Participants had diverse academic backgrounds. Notably, 12 (57.1%) hold master’s degrees (of them, 19% were OM-focused), 8 (38.1%) have doctoral degrees (4.8% in OM), and 8 (38.1%) hold specializations (of them, 9.5% in OM) (Table 1). Two (9.5%) respondents did not obtain formal postgraduate preparation in the field but claimed to have developed OM-based experience through years of clinical practice. Concerning international academic exposure, most respondents (76.2%) indicated international academic training (Supplement 3). Furthermore, 14 (66.7%) participants reported complementary interdisciplinary training (Supplement 4).

- Characterization of the specialty

Table 2 compiles questionnaire responses providing insights into OM practice in LAC, where most participants (66.7%) affirmed the official recognition of OM as a specialty within their respective countries. Year of recognition spanned from 1990 (Colombia) to 2016 (Paraguay). Responses varied regarding regulation, recognition, or authorization for OM specialty: 8 (38.1%) participants attributed this role to the Ministry of Health, 5 (23.8%) to the Ministry of Education, and 10 (47.6%) to faculties, associations, or federations. Fourteen (66.7%) participants reported disjunction of OM and OP in their countries, considering them as separate fields. Still, absence of standards, policies, or regulations to perform OM was reported by 10 (47.6%) participants.

National associations, federations or societies representing OM were recognized in only seven countries (33.3%); of these, 5 (71.4%) maintained a presence on social media platforms such as Facebook and/or Instagram, and 2 (15%) have scientific journals (Supplement 5). Quality assurance in OM practice was only reported by Ecuador, Chile, Colombia, and Mexico (19%), whereas for recertification exams of OM specialists, only Mexico (4.8%) confirmed their requirement. As for the definition of competencies or capacities for OM practitioners, 47.6% of participants indicated that competencies were well defined, as presented in Table 2.

- Academic training

Regarding academic training, 76.2% reported the absence of OM postgraduate courses in their countries. Among countries offering these courses, participants from Chile, Mexico, and Colombia (14.3%) reported having postgraduation programs combined with other dental areas (Supplement 6). In comparison, Brazil, and Venezuela (9.5%) reported that they were offered as a standalone field. Available postgraduate options included specializations (19%), master’s degrees (19%), doctorate degrees (4.8%), and no OM-focused residencies. Concerning government funding for academic OM training, Brazil, Chile, Costa Rica, and Mexico reported it, accounting for a 19% total coverage rate in the LAC region.

- Professional performance

The study identified diverse work opportunities for OM practitioners within their countries, including universities, hospitals, private practice, and research institutions: Nineteen (90.5%) participants stated private practice as the main national work field available for OM in their countries, followed by public and private universities with 18 (85.7%) positive answers each. The least reported work field available was research at private institutions, with 13 (61.9%) positive answers.

Participants estimated the number of certified OM specialists per country, and results were variable: a considerable amount of 11 countries (52.4%) reported 0-5 specialists, followed by Costa Rica, Ecuador, and Uruguay (14.3%) reporting 6-10, Venezuela and Nicaragua (9.5%) reporting 11-20, Argentina and Chile with 21-50 specialists, respectively. Brazil, Colombia, and Mexico (14.3%) stand out as countries reported to have over 100 specialists per country (Fig. 1). Responses varied regarding patient load, with 9 (42.9%) participants seeing fewer than 350 patients annually and 8 (38.1%) seeing between 351 and 700. Finally, OM practitioners from Bolivia and Panama (9.5%) reported seeing from 701 to 1,000, and respondents from Brazil and Colombia (9.5%) both see over 1,000 patients yearly. Monthly patient estimates were as follows: 11 countries (52.4%) had fewer than 30, 7 countries (33.3%) between 31 and 60, 1 (4.8%) ranging from 61 to 90, and 2 (9.5%) over 91 patients.

Our results about monetary compensation sources show that 15 (71.4%) participants receive remuneration from private financing or insurance, 14 (66.7%) from public funding or government agencies, and 1 (4.8%) from a specific financing agency, with some participants having multiple sources of income. Finally, the main identified barriers to OM practice were the need for recognition by multidisciplinary health teams (90.5%) and the low priority given to OM (71.4%).

## Discussion

This study assessed various aspects of OM in LAC, including sociodemographic, academic, educational, and professional dimensions. Previous investigations have examined OM in Europe and North America (2,3,18), and recently, a study evaluating professional training and practice of both OM and OP in 11 Latin America countries was published (14). Our approach represents a novel, comprehensive study assessing OM status across 21 countries in this distinct region, making it a crucial contribution of evidence for future global recommendations involving OM.

A key finding emerged during participant recruitment: it was not possible for this team to reach OM specialists in all LAC countries, even when broadening our criteria to include professionals without formal specialist training. As a result, we did not obtain information from certain nations, primarily those in the Caribbean, where linguistic differences and geographic separation from Hispanic-speaking continental countries may limit networking opportunities. Nonetheless, previous studies assessing OM globally reported participation from only 5 LAC countries (2), and those focused on Latin America also do not include Caribbean representation (14). In contrast, we included participants of almost two-thirds of the LAC region, marking the most representative sample of OM practitioners in the LAC region documented to date.

- Demographic aspects

Experts consulted showed a slight male predominance with a mean age of 46.9 years, similar to previous reports (7), but contrasting with other studies reporting a slight female predominance in a similar age range (3,19). Most professionals in our sample hold master’s degrees, while 38.1% have obtained PhDs. A lack of advanced clinical training in OM through residency hospital-based programs was also identified. This aligns with findings from previous studies highlighting the prevalence of master’s and doctoral degrees among OM experts (3,19), which represents a disparity between OM practitioners focused on clinical vs. research orientation that has been also already pointed out in literature (5).

Most participants (76.2%) received international academic training, compared to 22.5% reported in Europe and Australia (7). Notably, only 9.5% of our respondents currently work abroad, in contrast to 36.8% reported in the mentioned study (7). This may suggest a growing local workforce in LAC, despite the fact that national recognition is still in progress. Also, many (66.7%) engaged in complementary interdisciplinary education across various dental and medical disciplines. The relevance of medical experience and training for OM specialists is a crucial factor recognized by previous work (5). Fostering cross-learning and scientific collaborations may impact further OM practice and recognition (1,10), as well as enhance OM trainees and hospital medical teams’ inter-health professional cooperation, especially in oncology services. Still, this international and interdisciplinary training lacks standardization, raising concerns about the uniformity of preparation for OM professionals regarding medical matters, as formerly discussed by Baum and Scully (5).

- Characterization of the specialty

We identified national OM recognition by any local registering authority in 66.7% of LAC countries. Similar results have been reported elsewhere, with a previous study obtaining 68% of answers confirming national recognition of the specialty (2). Conversely, regional assessment in 11 countries of South and central America reported 90.9% of recognition of either OM or OP (15). In the Middle East, only 4 countries were reported to be recognized (20), and in Europa, only 3 (1). It seems worth noting that, from our results, a significant portion of positive responses about this matter reports regulation by dental schools or OM associations, so governmental regulation was often lacking. From this perspective, it is difficult to guarantee that academic requirements for OM performance and training are standardized. This is why the regulation of the specialty exclusively by universities or by independent societies does not seem to be sufficient.

Worldwide, the United States was the first to propose OM as a dental specialty in 1925, followed by Europe in the 1950s (21). Similarly, the creation of academies and associations in the field first occurred in the USA with the American Academy of Oral Medicine in the 1940s, while in Europe, possibly the first creation was the British Society of Oral Medicine in 1981 (1,21). Nonetheless, literature findings report a low number of OM societies worldwide (1). In our study, only a minority of countries had OM national associations or societies, indicating a need for greater professional support and regulation, as previously observed (4,12). Likewise, 47.6% of responders reported the absence of standards, policies, and regulations to perform OM. The need to establish practice standards and achieve calibration in this matter is crucial to ensure professional development to meet patient needs (22), and it has already been studied in the OM undergraduate program in the UK and Ireland (18). This finding can be extrapolated to the rest of the world, and it could possibly be facilitated by global collaboration through this national organizations.

Participants of the present study generally distinguished OM as an independent field from OP. This is not surprising, since the difference between the two areas is well understood by professionals in the field. However, dissimilar findings have been shown by previous reports, as European studies have reported that OM is usually combined with other dental disciplines, primarily OP (2,21), reflecting the complementary nature of these areas in providing comprehensive healthcare (20).

In LAC, OM practitioners predominantly found their professional engagement in private practice (90.5%) and academic activities at universities (85.7%), in agreement with former work in Latin America (14). The relevance of clinical practice (3) and academia (19,23) as a workplace for OM practitioners was shown previously. Conversely, Al-Amad. *et al* reported that only 16.6% of respondents worked in the private sector (19). These patterns may be influenced by local socioeconomic factors and the state of healthcare infrastructure, as reports in developed countries have had contrasting findings (8). The diversity in how OM competency is defined across the surveyed countries was also identified, a worldwide situation already reflected in former research (2,3,6,21,24) that underscore the pressing need for standardized competencies in the field to benefit both practitioners and patients (10).

- Academic training

A limited number of countries within the LAC region have postgraduate programs in OM, and some of these programs are combined with other specialties. Remarkably, no OM-focused hospital-based residency is currently available in LAC. This can be compared to the data gathered by Rogers. *et al*, in which 22 countries were reported as having postgraduate programs in OM but 9 of them were combined with another distinct field of study such as OP, oral radiology, and special care dentistry (2). In the Middle East, only 2 countries reported to have OM exclusive training programs, and other 2 in conjunction with OP (20). Nevertheless, lacking supranational coordination, the development of OM is pursuing dissimilar paths (7). Funding for OM training remained scarce, reflecting the disparities in financial support for OM trainees and senior professionals seen globally (3,19). The pursuit of robust international cooperation is a pivotal step towards comprehensive global guidelines for the practice of OM. As former proposals of advanced training curriculum have given limited consideration of LAC particularities (10), our findings hold significant value for future academic model development of OM framework (12) and emphasize the need for broader efforts to enhance OM education and training opportunities in LAC.

- Professional performance

There is limited information on the existing health workforce in Latin America and the Caribbean (13). Still, the global shortage of OM specialists and the dependence on non-specialists for OM care have been well-documented (18,19). Our data show a notably low prevalence of OM practitioners in the LAC region, especially in the Caribbean islands. This fact could have relevant impact in patient care and professional standards (25). In their assessment, Al-Amad. *et al* identified that 21% of respondents did not attend an OM graduate program (19). In the context of our survey, this gap was addressed by reaching out experienced academic dental professionals, leading to a lower value of 9.5%. These individuals, with an average of 18 years of practice in teaching and treating OM patients, provided valuable insights. Yet, the regional need of OM practitioners with academic and clinical preparation for OM is highlighted in agreement with the global panorama (1-3), and in this sense, the accessibility through digital approaches such as telemedicine could be of great benefit.

In addition, the recognized patient load was also low, reporting less than 700 cases per year and fewer than 30 patients per month per practitioner, a number that is certainly not due to a lack of patients with OM needs, as the literature clearly shows this population is, in fact, growing (6). Instead, it may reflect that the patients are not arriving to OM services, either due to their own lack of awareness of OM specialists, scarce referrals from dentists that are not specialized in OM, or because of undervaluing of the specialty by other health areas.

We showed that most OM practitioners rely on private practice activities to support their income, which can be explained by poor access to public health care in LAC as a significant struggle (13,26). Similar findings regarding Spain and Italy were already reported, where the lack of recognition, few job opportunities in the public health system and most often part-time employment in academic settings, unable an adequate income for most OM clinicians (7). This situation could reinforce the idea that exclusive dedication to OM is not enough to financially support a professional, which has been considered a barrier (4). Correspondingly, the most relevant reported barriers to perform OM practice were consistent, with a consensus on lack of recognition by the multidisciplinary health teams (90.5%) and low priority (71.4%) as the main difficulty.

Surveys have been proved to serve as suiTable tools for research, but they imply some biases. Despite efforts to ensure reliability, responses may still be influenced by variations in participants’ interpretation, knowledge, and perception. We also understand that the use of a non-probabilistic purposive sampling method, as done in cited similar studies, is a limitation, as a single representant per country cannot capture the full scope of all OM practitioners across LAC, and differences in perspectives among other colleagues are to be expected. However, as this area of study is clearly understudied, we believe that these initial approaches are a pivotal basis to set ground for further research. To minimize said factor, we aimed for representative OM practitioners that work in multiple public and private institutions and have a wide notion of OM national practice. Yet, we recognize the potential biases and restrictions regarding generalizability of our findings. Also, the nature of our sample did not allow for statistical analysis. Therefore, our data should be interpreted with caution.

In conclusion, this study provides a current view of the OM landscape regarding recognition, training, and practice in LAC, delineated by a group of 21 regional OM practitioners. Responses from this survey identified national OM recognition in two-thirds of LAC surveyed countries, and absence of professional performance regulations in almost half of them. Only 23.8% of LAC countries reported national OM postgraduate courses, without any OM-focused hospital-based residencies. As for professional practice, most respondents reported a patient load under 700 patients annually and stated private practice as their main work field. The data collected in this study, which show international variations in regulatory requirements, recognition, training, and practice, highlight the need to establish a clearer, standardized framework, and can serve as a basis for strategic actions to strengthen health systems. Further research involving more OM practitioners from both participant and non-participant countries, as well as studies assessing OM competencies and potential student interest in this field in LAC, is encouraged.

## Figures and Tables

**Table 1:** Participant’s demographic and academic information.

Characteristics		N (%)
Total		21 (100)
Gender	Female	10 (47.6)
	Male	11 (52.4)
Age (years)	Mean	46.9
	Range	32-76
Location	Current workplace at origin country	19 (90.5)
	Current workplace at different country	2 (9.5)
Academic background*: Specialization	Total Specialization	8 (38.1)
	Oral Medicine	2 (9.5)
	Oral and Maxillofacial Pathology	5 (23.8)
	Oral Medicine and Oral and Maxillofacial Pathology combined	1 (4.8)
	Other	0 (0)
Academic background*: Master's degree	Total Master's degree	12 (57.1)
	Oral Medicine	4 (19)
	Oral and Maxillofacial Pathology	6 (28.6)
	Oral Medicine and Oral and Maxillofacial Pathology combined	1 (4.8)
	Other	1 (4.8)
Academic background*: Doctorate's degree	Total Doctorate's degree	8 (38.1)
	Oral Medicine	1 (4.8)
	Oral and Maxillofacial Pathology	3 (14.3)
	Oral Medicine and Oral and maxillofacial pathology combined	0 (0)
	Other	4 (19)
Did not obtain formal postgraduate preparation on the field, but has developed experience through years of professional practice		2 (9.5)
International academic training	Yes	16 (76.2)
	No	5 (23.8)
Complementary interdisciplinary training	Yes	14 (66.7)
	No	7 (33.3)

**Table 2:** Survey responses regarding OM recognition, training, and practice in LAC.

Questionnaire			N (%)
National characterization	In your country, is the Oral Medicine specialty officially recognized by any local registering authorities?	Yes	14 (66.7)
		Mean (recognition year)	2007
		Range (years)	1990-2016
		No information about date	7 (33.3)
		No	5 (23.8)
		Does not know / Does not answer	2 (9.5)
	Who regulates / recognizes / authorizes the Oral Medicine specialty in your country? *	Ministry of Health	8 (38.1)
		Ministry of Education	5 (23.8)
		Faculties/Associations/Federations	10 (47.6)
		Does not know / Does not answer	1 (4.8)
		Other	0 (0)
	Is Oral Medicine an independent field from Oral and Maxillofacial Pathology?	Yes	14 (66.7)
		No	7 (33.3)
		Does not know / Does not answer	0 (0)
	Are there any standards / policies / regulations to perform Oral Medicine?	Yes	8 (38.1)
		No	10 (47.6)
		Does not know / Does not answer	3 (14.3)
	Which are the possible work fields available for Oral Medicine practitioners in your country? *	Public universities	18 (85.7)
		Private universities	18 (85.7)
		Public hospitals	17 (81)
		Private hospitals	14 (66.7)
		Public practice	14 (66.7)
		Private practice	19 (90.5)
		Research at public institution	17 (81)
		Research at private institution	13 (61.9)
		Other	0 (0)
	Are there any National Associations / Federations / Societies in your country that group Oral Medicine practitioners?	Yes	7 (33.3)
		No	13 (61.9)
		Does not know / Does not answer	1 (4.8)
	Are there activities of quality assurance of Oral Medicine practice carried out in your country to ensure high standard of performance regarding professional practice?	Yes	4 (19)
		No	16 (76.2)
		Does not know / Does not answer	1 (4.8)
	Do Oral Medicine specialists must do a recertification exam?	Yes	1 (4.8)
		No	16 (76.2)
		Does not know / Does not answer	4 (19)
	Are the competencies / capacities of the Oral Medicine practitioners defined?	Yes	10 (47.6)
		No	8 (38.1)
		Does not know / Does not answer	3 (14.3)
	Which areas are considered competencies of an Stomatologist / Oral Medicine practitioner in your country? *	HIV-related complications	18 (85.7)
		Other immunocompromised patients and their oral complications	17 (81)
		Complications related to cancer treatment	17 (81)
		Oral manifestations of systemic diseases	17 (81)
		Facial pain, neuralgia	13 (61.9)
		Sensory and taste disturbances	13 (61.9)
		Infections of the maxillofacial complex	12 (57.1)
		Oral management in patients in intensive care	10 (47.6)
		Dental care of the elder and/or polypharmacy patients	7 (33.3)
National characterization	Which areas are considered competencies of an Stomatologist / Oral Medicine practitioner in your country? *	Diseases of the dentin, pulp, and periodontal complex	7 (33.3)
		Dental care of transplant patients	7 (33.3)
		Dental care of patients with cardiovascular conditions	6 (28.6)
		Dental care of patients with chronic renal disease	6 (28.6)
		Dental care of patients with hemostasis disorders	6 (28.6)
		Dental care during pregnancy and lactation	5 (23.8)
		Dental care for patients with endocrine and metabolic disorders	5 (23.8)
		Dental care of patients with neurological and/or psychological/psychiatric disorders	5 (23.8)
		Dysfunction of the temporomandibular joint	5 (23.8)
		Other	9 (42.9)
Academic training	Are there Oral Medicine postgraduate courses in your country?	Yes	5 (23.8)
		No	16 (76.2)
		Does not know / Does not answer	0 (0)
	Is the Oral Medicine postgraduate course a unique field of study or is it combined with other area(s)?	Single	2 (9.5)
		Combined	3 (14.3)
		Does not apply	16 (76.2)
	What are the types of Oral Medicine postgraduate courses available in your country*	Specialization	4 (19)
		Master's degree	4 (19)
		Doctorate's degree	1 (4.8)
		Residency	0 (0)
	Type of Oral Medicine training and duration in months	Specialization	12-24
		Master's degree	24-36
		Doctorate's degree	40-60
		Residency	0
		Others (courses / training / diplomas)	0
	Are there any government funding resources for academic training in Oral Medicine in your country?	Yes	4 (19)
		No	14 (66.7)
		Does not know / Does not answer	3 (14.3)
	How many certified Oral Medicine specialists do you estimate that work in your country?	0-5	11 (52.4)
		6-10	3 (14.3)
		11-20	2 (9.5)
		21-50	2 (9.5)
		>100	3 (14.3)
Professional performance	How many patients are received at the Oral Medicine service you are linked to per year?	<350	9 (42.9)
		351-700	8 (38.1)
		701-1000	2 (9.5)
		>1000	2 (9.5)
	What is the estimated number of patients you see monthly? (Individual metric):	<30	11 (52.4)
		31-60	7 (33.3)
		61-90	1 (4.8)
		>91	2 (9.5)
	Where does the remuneration for the work you do comes from? *	Public financing / Government agencies	14 (66.7)
		Private financing / Consortia / Insurance	15 (71.4)
		Specific financing agencies	1 (4.8)
	Which of these do you consider to be the biggest barrier to professional performance in Stomatology / Oral Medicine in your country? *	Lack of knowledge and/or recognition by the multidisciplinary health team, including dentists	19 (90.5)
		Oral Medicine professional performance is considered of low priority	15 (71.4)
		Scarcity of available work positions	14 (66.7)
Professional performance	Which of these do you consider to be the biggest barrier to professional performance in Stomatology / Oral Medicine in your country? *	Professionally and/or financially, it is difficult to have exclusive dedication to this area	13 (61.9)
		Overlapping with other specialties	12 (57.1)
		Insufficient number of training programs	11 (52.4)
		Limited access to patients	7 (33.3)
		High number of Oral Medicine practitioners in the country in relation to the low demand of patients who require the service	0 (0)
